# The Desmosomal Plaque Proteins of the Plakophilin Family

**DOI:** 10.1155/2010/101452

**Published:** 2010-04-21

**Authors:** Steffen Neuber, Mario Mühmer, Denise Wratten, Peter J. Koch, Roland Moll, Ansgar Schmidt

**Affiliations:** ^1^Institute of Pathology, Philipps University of Marburg, Baldingerstraße, 35033 Marburg, Germany; ^2^Departments of Dermatology, Cell & Developmental Biology, University of Colorado Medical School, Aurora, CO 80045, USA

## Abstract

Three related proteins of the plakophilin family (PKP1_3) have been identified as junctional proteins that are essential for the formation and stabilization of desmosomal cell contacts. Failure of PKP expression can have fatal effects on desmosomal adhesion, leading to abnormal tissue and organ development. Thus, loss of functional PKP 1 in humans leads to ectodermal dysplasia/skin fragility (EDSF) syndrome, a genodermatosis with severe blistering of the epidermis as well as abnormal keratinocytes differentiation. Mutations in the human PKP 2 gene have been linked to severe heart abnormalities that lead to arrhythmogenic right ventricular cardiomyopathy (ARVC). In the past few years it has been shown that junctional adhesion is not the only function of PKPs. These proteins have been implicated in cell signaling, organization of the cytoskeleton, and control of protein biosynthesis under specific cellular circumstances. Clearly, PKPs are more than just cell adhesion proteins. In this paper we will give an overview of our current knowledge on the very distinct roles of plakophilins in the cell.

## 1. Introduction

Cellular adhesion is mediated by distinct protein complexes at the cytoplasmic membrane, termed junctions, that have been characterized by their morphology on the ultrastructural level [1]. Desmosomes reveal a characteristic appearance and anchor different types of intermediate filaments (IF) to the cell membrane. The fundamental functional importance of desmosomal cell contacts for cellular and tissue architecture, differentiation, development, and tissue stability is generally accepted and has previously been described [2–4]. Experimental evidence for the importance of desmosomal adhesion for specific tissues and organs has been established by knockout experiments of desmosomal genes in mice (see, e.g., [5]). Moreover, examination of a variety of human diseases characterized by a loss, or impairment of desmosomal adhesion—regardless of genetical, autoimmune, or infectious etiology—advanced our understanding of desmosomal function [6]. Desmosomes are formed by all epithelial tissues and tumors derived therefrom as well as by specific nonepithelial tissues such as heart muscle cells. Desmosomal cadherins (i.e., desmogleins DSGs and desmocollins DSCs) located on adjacent cells mediate intercellular connection via interactions of their extracellular domains (for review see [7]). On the cytoplasmic side of the plasma membrane, IF are linked to the desmosomal cadherins via desmosomal plaque proteins. Besides the constitutive desmosomal plaque proteins desmoplakin (DSP) and plakoglobin (JUP), at least one of the three classical members of the plakophilin family (PKP 1 to PKP 3) is required for the formation of functional desmosomes [8–10]. The role of PKPs in cellular adhesion have been analyzed in detail during the past decade [8–10]. However, additional functions of the plakophilins that are not directly linked to desmosomal adhesion have recently been described. In this review we want to provide insights not only into the known properties and functions of plakophilins in desmosomes, but also into cellular functions not related to adhesion.

## 2. Common Features of the Plakophilins

Plakophilins are probably the most basic proteins identified in cellular adhesion complexes so far with an isoelectric point (pI) of about pH 9.3. Based on their primary sequences, PKPs have been classified as a distinct subfamily of the armadillo repeat proteins (for review see [11]). The carboxyl-terminal part of the proteins includes nine armadillo repeats which contain a spacer sequence between the fifth and sixth repeat that leads to a characteristic kink in the domain structure as determined by crystallography of the armadillo domain of PKP 1 [12]. The amino-terminal parts (head domain) of the three plakophilins are rather diverse and exhibit no obvious homology to themselves or other proteins. Only a small sequence near the amino-terminus, designated homology region (HR) 2, shows some degree of homology between the plakophilins. An analysis of amino acid sequence homology reveals that the PKPs are related to the catenin proteins of the p120^ctn^-group, which are associated with classical cadherins, such as E-cadherin, in adherens junctions. The PKPs are more distantly related to the classical catenins, *β*-catenin and plakoglobin [8, 13]. PKPs show complex but overlapping expression patterns in mammalian tissues. Certain cells and tissues express only one type of PKP. Mutations affecting the corresponding PKPs thus can lead to severe diseases in these tissues since compensatory PKP isoforms are not expressed or may not substitute for all functional aspects. This probably explains the severe skin diseases caused by PKP 1 mutations and the heart diseases caused by PKP 2 mutations.

### 2.1. Plakophilin  1

**Figure 1:**
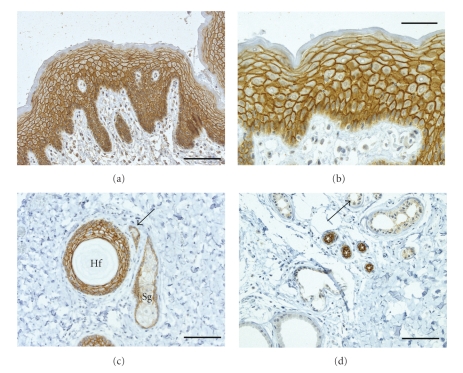
Immunohistochemical staining of sections of human skin with antibodies against PKP 1. Sections of formaldehyde-fixed tissue samples of human skin were stained with a monoclonal antibody (clone PP1 5C2; Progen, Heidelberg; for methods see [18]) against PKP 1 a to d. (a) Overview of epidermis showing a strong reaction of the antibodies at the desmosomes of all layers. (b) At a higher magnification, the basal layers exhibit a somewhat weaker desmosomal staining that can be resolved occasionally into individual spot-like desmosomes containing PKP 1. During keratinocyte differentiation, desmosomal labeling is getting more pronounced. (c) Cross-section of a hair follicle (Hf) with desmosomal staining of the outer root sheath while the hair-shaft is not stained (Sg, sebaceous gland). Arrow marks the duct of a sebaceous gland. (d) Eccrine sweat ducts are marked intensively by antibodies while the secretory portions of eccrine glands show a distinct but weaker staining (arrow). Apocrine sweat glands (lower left corner) are negative. Scale bars: 100 *μ*m (b); 200 *μ*m a, c, and d.

PKP 1 is the smallest of the plakophilins, with a calculated molecular weight of 80.497 Da and an apparent molecular weight of approximately 75 kDa as judged by SDS-PAGE [14]. This protein is localized in the desmosomes of stratified, complex, and transitional epithelia but is absent in simple epithelia [14–16]. In stratified epithelia, PKP 1 is synthesized in all cell layers, with an increase in expression from the basal to the granular compartment as determined by quantifications of PKP 1-specific immunofluorescence signal intensity in human epidermis [17]. This indicates that PKP1 is a marker for keratinocyte differentiation. PKP 1 appears to be absent in the *stratum corneum* of stratified squamous epithelia though (Figure 1).

The human PKP 1 gene is expressed as two different splice variants which differ with respect to cell-biological behavior, molecular weight, and abundance. PKP1a is the smaller isoform while the larger PKP1b isoform (predicted molecular weight: 82.860 kDa) is less abundant in stratified epithelia. The additional amino acid sequence contained in PKP1b is encoded by exon 7 which is spliced out of the PKP1a mRNA [19]. The PKP1b-specific amino acid sequence is located at the end of the fourth armadillo repeat and has a distinct effect on the cell biological activities of the protein. In addition to its desmosomal localization, PKP 1 has been detected in the nucleus of a broad range of cell types, even in those that do not incorporate PKP 1 in desmosomes such as simple epithelial cells [14, 19]. This distinct subcellular distribution has been observed for both variants of PKP 1. While the smaller PKP 1a may also be present in desmosomes, PKP 1b localization is restricted to the nucleus and not detectable in desmosomes. This conclusion is supported by transfection of cDNAs into cultured cells, where PKP 1a accumulates in desmosomes and is also rapidly transferred into the nucleus, while PKP 1b is only nuclear (own observations). Nevertheless, neither the way PKP 1 enters the nucleus nor the functions of this protein therein are yet known. 

Both the nuclear and desmosomal PKP 1 pool are degraded by caspases rapidly during apoptosis of keratinocytes suggesting that this protein is involved in the remodeling of the cytoskeleton under these conditions [20]. Signaling functions, as shown for some of the related catenins such as *β*-catenin, plakoglobin, and p120^ctn^, have been postulated for PKP 1, but proof is still lacking [21, 22]. A typical nuclear localization signal has not been identified in the protein so far, but cDNA transfection studies of the complete protein or individual parts of the protein into cells have shown that the head domain on its own, and to some extent the armadillo domain, are able to enter the nucleus [23]. The mechanism of the PKP 1 nuclear migration is currently unknown, but may utilize a piggyback mechanism.

Various in vitro approaches revealed that the binding of desmosomal PKP 1 to other desmosomal proteins such as DSP, DSG 1, DSC 1, and different keratins is mediated by its head domain sequence [24–28]. The armadillo repeat domain of PKP 1 alone is sufficient to localize the protein to the plasma membrane [23]. The PKP 1 binding partner at the plasma membrane has not been determined but might be one of the desmosomal proteins or even cortical actin. In particular, it has been observed that the armadillo domain coaligns with actin microfilaments under certain circumstances and may be involved in the reorganization of this cytoskeletal component [26]. Nevertheless, the carboxyl-terminal part, in particular the last 40 amino acids, seems to be essential for the recruitment of the entire PKP 1 to the plasma membrane as shown by transfection studies of mutant cDNA constructs into A431 keratinocytes [28].

**Figure 2:**
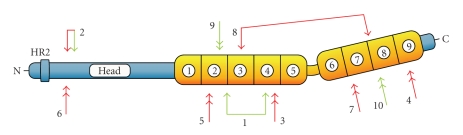
Position of mutations in human PKP 1 gene. Schematic representation of the protein structure of PKP 1 with head domain “Head” in blue color containing the homologous region 2 “HR2” near the amino-terminus which is followed by nine armadillo repeats (yellow boxes; numbered in circles from 1 to 9). Finally, a short domain (blue) at the carboxyl-terminus is shown. Positions of homozygous mutations are marked by double arrows, positions of compound heterozygous mutations by connected arrows. Green arrows designate mutations affecting the coding region, red arrows denote splice-site mutations. For numbering and references of the mutations see Table 1.

Important clues for the understanding of PKP 1 function came from a report of an autosomal-recessive genodermatosis that is caused by mutations in the PKP 1 gene [29]. The ectodermal dysplasia/skin fragility (EDSF) syndrome (OMIM 604536; the collection of known mutations in the PKP 1 gene is shown in Figure 2 and published cases of EDSF syndrome are listed in Table 1) clinically manifests in the skin and its appendages. Patients suffer from blistering with erosions of their skin upon mechanical stress. Nails are dystrophic and the epidermis of soles and palms displays hyperkeratosis. The hair density on the scalp, eyebrows, and eyelashes is reduced. In severe cases, hair might be completely absent from these body regions. Impaired sweating has occasionally been observed. All other epithelial tissues that express PKP 1, including mucous membranes, seem to be normal in these patients, suggesting functional compensation by the other PKPs. Histological examination of affected skin reveals that the intercellular space is widened and epidermal keratinocytes are acantholytic from the suprabasal layers upwards, suggesting loss of cell-cell adhesion. Cell rupture, as noticed for epidermolytical bullous dermatosis, has not been observed. Immunofluorescence microscopy analyses of patients' skin biopsies showed that certain desmosomal components such as desmogleins, desmocollins, and plakoglobin are still localized at the plasma membrane. In contrast, PKP 1 is completely absent or drastically reduced [30]. As a consequence, desmoplakin is no longer localized in the desmosomes but instead is dispersed throughout the cytoplasm. On the ultrastructural level, desmosomes appear smaller and are numerically reduced in the affected epidermal layers. Additionally, keratin filaments have lost contact to desmosomal junctions and are collapsed around the nucleus. Biochemical analysis of patients' skin revealed that the other PKPs are upregulated to some extent and may compensate in part for the loss of PKP 1 in nonaffected epidermal layers [17]. Interestingly, it does not seem to matter for the development of the clinicopathological findings of EDSF syndrome to what extent the protein is truncated due to the mutations in PKP 1 gene. In a case reported by McGrath and colleagues the mutations occurred close to the amino-terminus of the protein, which could result either in a severely truncated protein or—more likely—in complete loss of the protein (i.e., a functional null mutation) as judged by immunofluorescence microscopy [29]. In contrast, the mutations in the PKP 1 gene reported by Hamada et al. occurred near the carboxyl-terminus resulting in the expression of a truncated protein. Based on the mild phenotype of the ESDF syndrome in these patients, it can be assumed that this truncated protein is at least partially functional but clinicopathology of ESDF still manifests [30]. Surprisingly, most of the EDSF-related mutations in human PKP 1 gene involve splice-site mutations (8 out of 13 known mutated alleles) leading to impaired splicing products and subsequent mRNA degradation or the generation of truncated proteins. The reason for the prevalence of splice-site mutations in EDSF is not known.

These findings in conjunction with cell biological data obtained in transfection studies convincingly illustrate that PKP 1 is essential for the recruitment of desmoplakin to the desmosomal plaque and probably is involved into lateral enlargement of the plaque structure in skin, explaining the structural and functional defects in epidermal desmosomes lacking PKP 1. Evidently, integration of PKP 1 in the desmosomes provides the epidermal keratinocytes with stability against mechanical stress. A sequence stretch in the HR2 domain of PKP 1 is thought to be essential for the recruitment of DSP and represents a conserved motif of all the PKPs, suggesting that DSP recruitment is a common function of all PKPs [28]. 

Although a direct interaction of PKP 1 with keratins has been demonstrated frequently in vitro, it is not clear whether this protein alone is sufficient to connect the intermediate filament cytoskeleton to the desmosome. Specific inactivation of DSP in the skin of mice demonstrates the necessity of both proteins, DSP and PKP 1 (in cooperation with plakoglobin), for anchorage of keratins [38] suggesting that all three components are required. This is further demonstrated by the fact that failure of either PKP 1 or DSP can lead to loss of cell-cell adhesion and acantholysis in the epidermis. The mechanism underlying the failure of epidermal desmosomes without PKP 1 to maintain adhesion is not known. It is tempting to speculate that besides structural defects cell signaling defects could contribute to this phenomenon, similar to the disease mechanisms postulated for the autoimmune blistering diseases of the *pemphigus* group in which autoantibodies target desmosomal cadherins. Binding of autoantibodies to the desmosomal cadherins seems to trigger intracellular signaling pathways that lead to the reorganization of the cytoskeleton involving the disconnection of desmosomal cadherins of adjacent cells (for the mechanisms of this outside-in signaling see [39]). The same pathways may be involved in the dissolution of desmosomal adhesion when PKP 1 is lost. Given that patients with PKP 1 null mutations show defects in differentiation pathways affecting skin appendage formation and homeostasis, it is unlikely that adhesion defects can account for the entire spectrum of disease phenotypes. 

Analysis of keratinocytes derived from patients suffering from EDSF syndrome exhibits some interesting properties. Quantitative analyses of the desmosome size in cultured cells revealed that reintroduction of PKP 1 increases the lateral extent of desmosomes. As proposed by others [25, 40], desmosomal cohesiveness might be increased by lateral interactions of PKP 1 with DSP, making additional linkage between desmosomal proteins and keratin network accessible [41]. It is noteworthy that PKP 1 null keratinocytes show increased cell migration, which has implications for tumor biology.

### 2.2. Plakophilin  2

**Figure 3:**
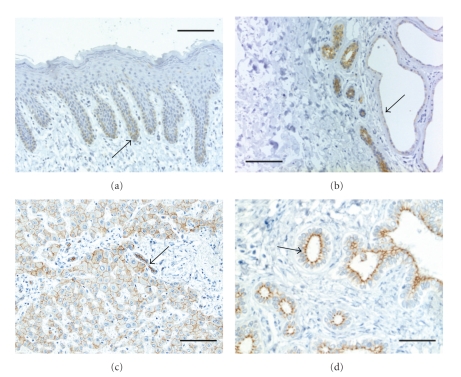
Immunohistochemical staining of sections of human skin a, and b and liver c, and d with antibodies against PKP 2. (a) The staining of samples of human skin with a monoclonal antibody against PKP 2 (clone PP2-150; Progen, Heidelberg) demonstrates a weak and delicate desmosomal staining as well as cytoplasmic staining in the basal layer of the interfollicular epidermis (arrow). Suprabasal keratinocytes remain unstained. (b) Eccrine sweat glands and ducts show a strong reaction with PKP 2-specific antibodies while apocrine sweat glands exhibit an apical, distinct but weak desmosomal reaction (arrow). (c) Hepatocytes as well as bile ductules are marked at the cell-cell contacts by PKP 2-specific antibodies (arrow). (d) Bile ducts also show a sharp and apical staining of desmosomal structure by the PKP 2-antibodies. The samples shown in (c) and (d) are derived from liver tissue in the vicinity of a metastasis of a gastrointestinal stromal tumor with portal and periportal fibrosis and ductal and ductular proliferation. Scale bars: 100 *μ*m (d), 200 *μ*m (a, b, c).

PKP 2 is, with a predicted mass of 92.756 Da and an apparent molecular weight of 100 kDa (estimated from Western blot analysis), the largest of the three plakophilins and it is also the prevailing isoform since it is expressed in all cell types with desmosomal junctions [42]. PKP 2 is found in the basal cells of certain stratified epithelia while more differentiated keratinocytes are negative for desmosomal PKP 2 (Figure 3). Moreover, PKP 2 has recently also been found in new types of cell junction which differ in terms of their biochemical composition from both classical desmosomes and conventional adherens junctions (reviewed in [43]). Similar to PKP 1, PKP 2 also occurs as two different splice variants. An additional exon coding for 44 amino acids is integrated into PKP 2 b close to the border of the second to third armadillo repeat of the protein [42]. The two PKP 2 splice variants appear to be coexpressed in all cell types analyzed thus far, and it is not known whether these two proteins have different functions.

Like PKP 1, PKP 2 has been detected in the nucleus of many cell types [42]. Its presence in the nucleus is independent of its presence in desmosomes. Some nonepithelial cell types, which do not assemble desmosomes, show only nuclear localization of PKP 2 (e.g., fibroblasts [42]). In stratified epithelia, nuclear and desmosomal localization of PKP2 is regulated independently. In the differentiated layers of stratified epithelia, PKP 2 is excluded from desmosomes and accumulates in the nuclei of keratinocytes. Recently, Müller and colleagues identified a molecular pathway that appears to regulate nuclear accumulation of PKP 2 [44]. The Cdc25C-associated kinase 1 (C-TAK 1) emerges to be involved in cell-cycle regulation and Ras-signaling. It was shown that C-TAK 1 phosphorylates Cdc25C and KSR1, a scaffold protein for mitogen-activated protein kinase (MAPK) and Raf-1 kinase. Müller et al. demonstrated that PKP 2 is also a substrate for C-TAK 1 [44]. This phosphorylation of PKP 2 enforces an interaction of PKP 2 with 14-3-3 proteins, which prevents the nuclear accumulation of PKP 2. Consequently, mutation of the C-TAK 1 phosphorylation site or the 14-3-3 binding domain in PKP 2 increases nuclear accumulation of PKP 2. The pathways that trigger C-TAK 1-mediated phosphorylation of PKP 2 and its retention in the cytoplasm have not been analyzed so far. 

What does PKP 2 do in the nucleus? Recent experiments by Mertens and colleagues provided some insights [45]. Immunoprecipitation experiments revealed an association of PKP 2 with the largest subunit of RNA-polymerase-III holoenzyme, protein RPC155, as well as other components such as RPC82 and RPC39. The PKP 2-positive complexes also contain RNA-polymerase-III-associated transcription factor TFIIIB but not TFIIIC. The colocalization of PKP 2 and RPC155 in particles in the interchromatin space has been shown by immunofluorescence microscopy. Mertens and colleagues [45] postulated that these particles do not represent active forms of polymerase-III, because the PKP 2-positive particles do not contain transcription factor TFIIIC, a factor required for the formation of an active RNA polymerase III complex. Thus, the actual function of these complexes remains unclear. Nevertheless, the almost general appearance of PKP 2, as well as PKP 1, in the nucleus seems to differ fundamentally from the nuclear localization of other related catenins such as *β*-catenin or p120^ctn^, which are translocated into the nucleus upon specific signals and have been shown to be involved in gene regulation [21, 22]. 

Besides these nuclear functions, PKP 2 may be involved in cytoplasmic signaling, which is based on the observation that it can bind *β*-catenin [46], a key downstream effector protein of the canonical Wnt-signaling pathway [21]. Using two-hybrid and immunoprecipitation assay, it was shown that PKP 2 can bind to *β*-catenin. However, when bound to PKP 2, *β*-catenin cannot associate to E-cadherin, which may reduce the pool of *β*-catenin available to function in cell adhesion. Overexpression of PKP 2 in colon carcinoma cells leads to an increase in *β*-catenin/TCF signaling suggesting a regulatory role of PKP 2 in Wnt signaling and providing a potential functional link between desmosomal adhesion and signaling [46]. 

PKP 2 also seems to be involved in the assembly of the desmosomal components into desmosomes. siRNA-mediated depletion of PKP 2 in keratinocytes leads to changes in the subcellular localization of DSP which mimics the behavior of a DSP mutant deficient for a PKC*α* (i.e., protein kinase C) phosphorylation site. Different isoforms of PKC have been implicated in the regulation of cellular processes such as migration, cellular adhesion, or cytoskeletal reorganization (for review see [47]). Bass-Zubeck et al. investigated the connection between PKP 2, DSP, and PKC [48]. The authors found that PKP 2 binds to PKC*α* and DSP via its head domain. A detailed analysis revealed that PKP 2 simultaneously binds DSP and PKC*α*, which facilitates the subsequent phosphorylation of DSP at its IF-binding domain by PKC [48]. This increases DSP integration into the desmosomes and the subsequent attachment of IFs to desmoplakin.

Insights into the function of PKP 2 also came from gene knockout experiments in mice, as well as the analysis of an autosomal-dominant human hereditary disease linked to PKP 2 mutations [49, 50]. Ablation of the PKP 2 gene in mice leads to a lethal phenotype around mid-gestation (E10.5) [49]. Homozygous PKP 2-null embryos died because of severe alterations of the heart structure resulting in the outflow of blood into the pericardium and subsequent collapse of the embryonic blood circulation. On the microscopic level, PKP 2 deficient hearts display reduced trabeculation as well as abnormally thin cardiac walls. The reason for the instability of cell contacts between cardiomyocytes is apparent on the ultrastructural level. The junctional complexes of the* areae compositae *(formerly designated as intercalated disks; see [43]) that connect cardiomyocytes include at least two types of junctions in an amalgamated fashion, desmosomes and adherens junctions. The *areae compositae* are altered significantly in PKP 2-mutant mice. Associated with the deficiency of PKP 2, DSP is depleted from the desmosomal junctions and accumulates in the cytoplasm. Additionally, DSG 2 expression seems to be reduced in PKP 2-null cardiomyocytes and desmosomal components were less resistant to detergent extraction, suggesting impaired function of cell junctions. Therefore, PKP 2 seems to be essential for the regular subcellular distribution of desmoplakin and its accumulation in the *areae compositae* of cardiomyocytes. Interestingly, Grossmann et al. found no alteration in other PKP 2-expressing epithelia in the mutant animals [49]. This is likely due to the expression of multiple PKP isoforms in many cell types (except for the heart which expresses only PKP 2), providing functional compensation in case one isoform is not functional.

The essential function of PKP 2 in the heart was also demonstrated by the identification of a haplo-insufficiency of PKP 2 in a hereditary human disease, autosomal-dominant arrhythmogenic right ventricular cardiomyopathy (ARVC; [50]). In ARVC, cardiomyocytes are progressively replaced by fibro-fatty tissue, especially in the right ventricle (for a recent review see [51]). This replacement leads to abnormal electrical conductance with syncopes and tachycardia and an often lethal failure in the mechanical capability of the heart (e.g., “sudden cardiac death” of young athletes). The mechanism leading to ARVC may include apoptosis of cardiomyocytes due to the weak and disrupted intercellular adhesion of cardiomyocytes caused by haplo-insufficiency of PKP 2 and subsequent insufficient anchorage of DSP [52]. The decline of cardiomyocytes may therefore lead to the development of scar tissue in the right ventricle. Moreover, transdifferentiation of cardiomyocytes into fibro- or adipocytes may take place, probably caused by disturbed Wnt/*β*-catenin-signaling [53, 54]. This is supported by further observations. The decrease of DSP in cultured atrial myocytes by siRNA results in the redistribution of plakoglobin to the nucleus and the suppression of the canonical Wnt/*β*-catenin-signaling pathway [54]. Genes inducing adipogenesis and fibrogenesis were upregulated in these DSP-deficient cells. Decrease of DSP was also noticed in cardiomyocytes of PKP 2-deficient mice [49], suggesting that a cellular transdifferentiation may also occur in ARVC. At least 12 different genes or chromosomal loci have been associated with the autosomal-dominant or recessive types of ARVC so far, including all five known desmosomal genes expressed in cardiomyocytes (i.e., DSG 2, DSC 2, DSP, JUP, and PKP 2).

The loss of PKP 2 may also contribute to the abnormal electrical conductance of the heart [55]. Gap junctions play an essential role in the electrical coupling of cardiomyocytes and the coordinated heart contraction (reviewed in [56]). Downregulation of PKP 2 in primary cardiomyocytes of rat heart leads to reduced expression of the gap junction protein connexin 42. In addition, a decrease of cellular coupling via gap junctions is also detectable, which may result in the disturbed transmission of electrical impulses in the ventricle. Therefore, it appears that PKP 2 can influence the organization of different types of cellular junctions such as gap junctions and* areae compositae *in heart muscle cells.

### 2.3. Plakophilin  3

**Figure 4:**
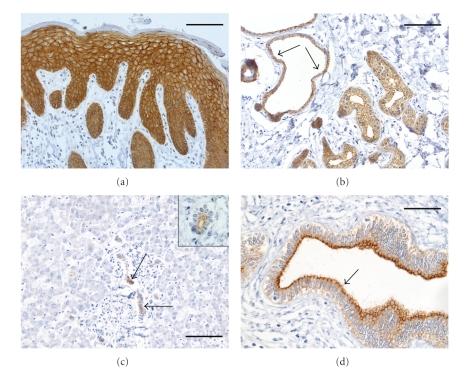
Immunohistochemical staining of sections of human skin a, and b and liver c, and d with antibodies against PKP 3. (a) Intensive reaction of desmosomes and cytoplasm is visible by staining sections of human skin with a monoclonal antibody against PKP 3 (clone PKP3 310.9.1; Progen, Heidelberg). Basal and lower suprabasal keratinocytes exhibit a strong cytoplasmic staining while desmosomal staining is less prominent. With ongoing differentiation, the desmosomal labeling is increasing. (b) Eccrine and apocrine (arrows) sweat glands show strong desmosomal labeling with PKP 3-specific antibodies. (c) Reaction of PKP 3-specific antibodies on liver is restricted to bile ductules (arrow; see description of liver tissue in the legend to Figure 3) while hepatocytes are completely negative for PKP 3. The insert presents a magnification of a bile ductule of human liver stained with antibodies against PKP 3, exhibiting a labeling of the desmosomal junctions. (d) Bile ducts (here in a large portal field) show a clear desmosomal reaction at the apical pole of cells (arrow). Scale bars: 100 *μ*m (d), 200 *μ*m a, b, c.

PKP 3 has a calculated mass of 87,081 Da and is detected with an apparent molecular weight of approximately 87 kDa on Western blot analysis [57, 58]. Strikingly, in contrast to the other PKP gens, PKP3 gene seems not to encode for different splice variants. PKP 3 is present in the desmosomes of all cell layers of stratified epithelia and in almost all simple epithelia, with the exception of hepatocytes (Figure 4). In epidermal cells, PKP 3 is expressed in a homogeneous pattern. Furthermore, it is detectable in the desmosomes of some nonepithelial cells with the notable exception of cardiomyocytes. This fact may explain the severe heart phenotype of PKP 2 loss, since PKP 2 is the only PKP expressed in cardiomyocytes and its loss of function cannot be compensated by the other PKPs. Although PKP 3 is mainly located in desmosomes, a significant proportion of the protein remains soluble in the cytoplasm. In contrast to the other PKPs, PKP 3 has not been detected in the nucleus.

A better understanding of the functions of PKP 3 came from the analyses of PKP 3 knockout mice [59]. In contrast to the other two PKPs, the PKP 3 knockout phenotype is fairly mild. PKP 3-null animals are viable and exhibit defects in the morphogenesis and morphology of specific hair follicles. Moreover, alterations in density and spacing of desmosomes and adherens junctions in PKP 3-null epidermis and oral cavity were observed (own unpublished observations). Consequently, PKP 3 is involved in the development or maintenance of skin appendages. Other PKP 3-positive epithelia appear normal in PKP 3-null animals. In addition, an upregulation of the expression of specific junctional proteins, such as the other PKPs, was noticed. In comparison to the other two PKPs, the PKP 3 knockout phenotype is modest, which may in part be due to the fact that an additional PKP is coexpressed in most epithelia and may compensate for at least some of the PKP 3 functions. Diseases in associated with the loss or heterozygosity of PKP 3 have not been reported so far.

Surprisingly, among the three plakophilins, PKP 3 exhibits the most extensive binding repertoire to other desmosomal components [60] and it demonstrates* in silico* the most extensive interaction rate of desmosomal proteins, as predicted for keratinocytes by Cirillo and Prime [61]. It is capable to bind to most of the desmosomal proteins such as all DSG and DSC isoforms, JUP and DSP and furthermore, it is the only PKP that interacts with the smaller DSC-b isoforms that are missing the binding site for plakoglobin [60]. This implicates an apparent binding site for PKP 3 at the juxtamembrane domain of desmosomal cadherins. Both the PKP 3 head domain and the arm-repeats seem to be crucial for these interactions, since most of the interactions to other desmosomal proteins occur in yeast two-hybrid assay only using the entire PKP 3 but not using the individual domains [60].

Further PKP 3 interaction partners are emerging, that are not linked to cell adhesion, suggesting a broader biological role of PKP 3. PKP 3 has been shown, for example, to interact with RNA-binding proteins such as poly-A binding protein C1 (PABPC1), FXR1 (Fragile X mental redartation-1), and G3BP (GAP SH3 domain-binding protein) in stress granules [62]. Stress granules develop when cells respond to diverse environmental stress conditions and these particles represent stalled translational complexes (for a recent review of stress granules see [63]). The function of PKP 3 in stress granules and the basis for the integration into stress granules remain unclear, but it seems likely that this is not a general function of all PKPs, since in addition to PKP 3, only PKP 1 but not PKP 2 has the ability to integrate into the stress granules.

Another PKP 3-binding protein identified is dynamin-like protein DNM-1L [64]. DNM-1L is involved in the peroxisomal and mitochondrial fission and fusion as well as mitochondrial-dependent apoptosis of cells [65, 66]. Although the biological significance for this interaction is not clear, it is tempting to speculate that the PKP 3 could affect the apoptotic response of cells.

### 2.4. Plakophilins in Tumors

Cellular adhesion molecules, especially components of the adherens junctions such as E-cadherin and *β*-catenin, have been shown to be important in the development, progression, and metastasis of tumors [67]. Likewise, several desmosomal proteins have also been linked to malignant processes (reviewed in [68]). Reliable data demonstrating a causal link between plakophilins and tumor development are still forthcoming. Thus far, most published studies focused on the expression of PKPs in tumors and a correlation of PKP expression and tumor prognosis. Well and moderately differentiated squamous cell carcinomas (SqCC) of skin express PKP 1, whereas in poorly differentiated tumors, PKP 1 is downregulated [69]. Tumor cells of basal cell carcinomas (BCC) exhibit a more heterogeneous expression of PKP 1, being confined to small patchy areas [69]. In solid nodular BCCs, PKP 1 expression has been found to be reduced in comparison to normal overlaying epidermis and was hardly detectable in nodules growing close to the basal epidermis. Immunohistochemical analysis of the expression of PKP 1 in oral SqCCs revealed similar results to those obtained with skin tumors [18, 70]. This is, however, conflicting with observations made by others [71], who found that PKP 1 is strongly expressed only in a small proportion of well-differentiated SqCCs. Furthermore, these authors found that most of the well-differentiated tumors are negative for PKP 1. Interestingly, using cells derived from oral SqCCs, Sobolik-Delmaire et al. [70] could demonstrate that cell lines expressing low levels of PKP 1 exhibit increased cell mobility which is reduced by ectopic expression of PKP 1. In contrast, another cell line of an oral SqCC that expresses comparably high levels of PKP 1 becomes more mobile and invasive in vitro when PKP 1 is diminished by a shRNA knock-down approach. 

Interestingly, in a part of oral and pharyngeal SqCCs analyzed by Schwarz et al. [18], nuclear localization of PKP 1 in tumor cells was noticed. This is remarkable since adjacent non-neoplastic squamous epithelium did not show nuclear PKP 1. In contrast to PKP 1, immunostaining for PKP 2 in histological sections of SqCC is low and often restricted to peripherally located tumor cells or is even completely absent [18], whereas PKP 3 expression patterns are similar to PKP 1 in SqCC. The expression of PKP 3 seems to correlate inversely with the degree of malignancy of tumors. 

An analysis of adenocarcinomas from different organs such as colon and pancreas revealed that PKP 1 is not detected whereas PKP 2 and PKP 3 are frequently expressed [18, 72], sometimes associated with a change from an apical desmosomal staining to a staining of almost complete lateral surface. The only exceptions were prostate adenocarcinomas which displayed a low level of PKP 1 immunoreactivity. Interestingly, in non-small cell lung carcinomas (NSCLC; adenocarcinomas and SqCC) and cultured cells derived thereof, Furukawa et al. observed an elevated expression of PKP 3 [64]. Inhibition of PKP 3 expression by siRNA approach in NSCLC cultured cells led to reduced colony formation and less viability of cells. Moreover, over-expression of PKP 3 in COS-cells caused enhanced proliferation rate and elevated activity in in vitro invasion assay. The authors postulated that PKP 3 may have an oncogenic function when localized in the cytoplasm under certain conditions. It thus appears that PKP 3 can potentially both, advance tumorigenesis (as seen in some NSCLC) or suppress it (as noticed for some SqCCs). Recent observations suggest that PKP 3 may be involved in epithelial-mesenchymal transition (EMT) that is of relevance especially for metastasis of tumor cells [73]. Analysis of PKP 3 expression in invasive cancer cells revealed that PKP 3 expression seems to be repressed by the transcription factor ZEB 1, a potent repressor of E-cadherin expression that is also involved in EMT, at least in breast cancer cells. Nuclear accumulation of ZEB 1 (i.e., Zinc finger E-box-binding homeobox-1) correlated with a loss of membrane staining for PKP 3. Similar observations have been reported for PKP 2-repression by ZEB 2 in colon cancer cells [74]. In conclusion, the precise role of PKPs in tumor development and tumor progression is not clear. It is possible that some of these proteins can function both, as oncogenes or as tumor suppressors, depending on the cell type studied. Further research is needed to establish a causal link between PKP expression (or loss of expression) and cancer.

In summary, in the past few years PKPs have been recognized to be essential for desmosomal adhesion and tissue integrity. Nevertheless, recent data suggest that PKPs exert cellular functions unrelated to cell adhesion. Further questions like the ability of individual PKPs to compensate for the loss of one isoform and the role of PKPs in cell signaling and in tumor development need to be further investigated.

## Figures and Tables

**Table 1:** Published cases of EDSF syndrome with clinical features and observed mutations in PKP 1 gene.

Case^1^	Clinicopathological findings					Observed mutations^2^	Reference
	Epidermal fragility	Hyperkeratosis on palms/soles	Alopecia	Nail dysplasia	Hypohidrosis		
1	yes	yes	yes	yes	yes	(a) p.Q304X (b) c.1132ins28	[29]
2	yes	yes	yes	yes	yes	(a) p.Y71X (b) IVS1−1G>A	[31]
3	yes	yes	yes	yes	no	IVS6−2A>T	[32]
4	yes	yes	yes	yes	yes	IVS11+1G>A	[30]
5	yes	no	yes	yes	no	IVS4−2A>G	[33]
6	yes	yes	yes	yes	not observed	IVS1−1G>A	[33]
7	yes	yes	no	yes	no	IVS9+1G>A	[34]
8	yes	yes	yes	yes	no	(a) c.1053T>A +IVS5+1G>A (b) IVS10−2G>T	[35]
9	yes	yes	yes	yes	no	c.888delC	[36]
10	yes	yes	yes	yes	not observed	p.R672X	[37]

^1^ Numbering of the case correlates to the positions of mutations shown in Figure 2.

^2^ For compound heterozygosity, mutations of both alleles are given as (a) and (b).
